# Nicotianamine Synthase 2 Is Required for Symbiotic Nitrogen Fixation in *Medicago truncatula* Nodules

**DOI:** 10.3389/fpls.2019.01780

**Published:** 2020-01-30

**Authors:** Viviana Escudero, Isidro Abreu, Eric del Sastre, Manuel Tejada-Jiménez, Camille Larue, Lorena Novoa-Aponte, Jorge Castillo-González, Jiangqi Wen, Kirankumar S. Mysore, Javier Abadía, José M. Argüello, Hiram Castillo-Michel, Ana Álvarez-Fernández, Juan Imperial, Manuel González-Guerrero

**Affiliations:** ^1^Centro de Biotecnología y Genómica de Plantas (UPM-INIA), Universidad Politécnica de Madrid, Madrid, Spain; ^2^EcoLab, Université de Toulouse, CNRS, Toulouse, France; ^3^Department of Chemistry and Biochemistry, Worcester Polytechnic Institute, Worcester, MA, United States; ^4^Estación Experimental de Aula Dei, Consejo Superior de Investigaciones Científicas, Zaragoza, Spain; ^5^Noble Research Institute, Ardmore, OK, United States; ^6^ID21 Beamline, European Synchrotron Radiation Facility, Grenoble, France; ^7^Instituto de Ciencias Agrarias, Consejo Superior de Investigaciones Científicas, Madrid, Spain; ^8^Escuela Técnica Superior de Ingeniería Agronómica, Alimentaria y de Biosistemas, Universidad Politécnica de Madrid, Madrid, Spain

**Keywords:** iron, metal homeostasis, nicotianamine synthases, nodulation, metal nutrition

## Abstract

Symbiotic nitrogen fixation carried out by the interaction between legumes and diazotrophic bacteria known as rhizobia requires relatively large levels of transition metals. These elements are cofactors of many key enzymes involved in this process. Metallic micronutrients are obtained from soil by the roots and directed to sink organs by the vasculature, in a process mediated by a number of metal transporters and small organic molecules that facilitate metal delivery in the plant fluids. Among the later, nicotianamine is one of the most important. Synthesized by nicotianamine synthases (NAS), this molecule forms metal complexes participating in intracellular metal homeostasis and long-distance metal trafficking. Here we characterized the *NAS2* gene from model legume *Medicago truncatula*. MtNAS2 is located in the root vasculature and in all nodule tissues in the infection and fixation zones. Symbiotic nitrogen fixation requires of *MtNAS2* function, as indicated by the loss of nitrogenase activity in the insertional mutant *nas2-1*, phenotype reverted by reintroduction of a wild-type copy of *MtNAS2*. This would result from the altered iron distribution in *nas2-1* nodules shown with X-ray fluorescence. Moreover, iron speciation is also affected in these nodules. These data suggest a role of nicotianamine in iron delivery for symbiotic nitrogen fixation.

## Introduction

Nitrogen is one of the main limiting nutrients in the biosphere, in spite of N_2_ abundance (Smil, 1999; Hoffman et al., 2014). Nitrogenase is the only enzyme that can convert, fix, N_2_ into NH_3_ under physiological conditions, in an energy consuming process (Burk, 1934; Burgess and Lowe, 1996). This enzyme is only expressed by the small group of diazotrophic archaea and bacteria, some of them participating in symbiosis with other organisms (Boyd and Peters, 2013). Arguably, one of the best characterized symbiosis with diazotrophic bacteria is the one established between rhizobia and legumes (Brewin, 1991; Downie, 2014). This symbiosis is the basis for legume use in crop rotation strategies and their potential as an alternative to polluting and expensive synthetic nitrogen fertilizers (Johnson and Mohler, 2009; Mus et al., 2016).

Symbiotic nitrogen fixation by the legume-rhizobia system is carried out in root nodules (Downie, 2014). These are differentiated organs that develop after a complex exchange of chemical signals between the symbionts (Oldroyd, 2013). Detection of the nodulation factors released by the rhizobia, triggers cell proliferation in the pericycle-inner cortex of the root to originate nodule primordia (Xiao et al., 2014). As nodules grow, rhizobia from the root surface are directed by infection threads to the nodule cells (Gage, 2002). There, they are released in an endocytic-like process, originating pseudo-organelles known as symbiosomes (Roth and Stacey, 1989; Catalano et al., 2006). Within the symbiosomes, rhizobia differentiate into bacteroids and express the enzymatic machinery required for nitrogen fixation (Kondorosi et al., 2013). Nodule development follows either an indeterminate or a determinate growth pattern, based on whether they maintain an apical meristem to sustain growth (Vasse et al., 1990). As this meristem allows for sustained growth in indeterminate nodules, four developmental zones appear: the meristematic region or zone I; the infection-differentiation zone or zone II, where rhizobia are released in the cell and start differentiating; the fixation zone or zone III, where nitrogenase is active; and the senescent zone or zone IV, where symbiosomes are degraded and nutrients recycled (Burton et al., 1998). In addition, some authors define a transition interzone between zones II and III (Roux et al., 2014).

Nutrient exchange between the symbionts enables nitrogen fixation (Udvardi and Poole, 2013). Availability of fixed nitrogen forms in soils inhibits nodulation (Streeter, 1987). Similarly, low levels of photosynthates, phosphate, or sulphate transfer from the host plant decrease nodulation and nitrogen fixation rates (Singleton and van Kessel, 1987; Valentine et al., 2017; Schneider et al., 2019). Transition metals such as iron, copper, zinc, or molybdenum are also critical for nodulation and nitrogen fixation as cofactors in many of the involved enzymes (González-Guerrero et al., 2014; González-Guerrero et al., 2016). This includes not only nitrogenase (Rubio and Ludden, 2005), but also nicotinamide adenine dinucleotide phosphate (NADPH)-oxidases that participate in nodule signaling (Montiel et al., 2016), leghemoglobin that maintains nodule O_2_ homeostasis (Appleby, 1984), high-affinity cytochrome oxidases providing energy to the bacteroids (Preisig et al., 1996), as well as many enzymes involved in free radical control (Dalton et al., 1998; Santos et al., 2000; Rubio et al., 2007). Consequently, deficiencies in the uptake of these nutrients or alterations in the metal delivery pathways lead to defects in nodulation and/or nitrogen fixation (Tang et al., 1991; O'Hara, 2001; Senovilla et al., 2018; Gil-Díez et al., 2019).

To reach the bacteroids, metals must first cross from soil into the roots using the general mechanisms common to all dicots (Kobayashi and Nishizawa, 2012; Curie and Mari, 2017). Metal uptake is facilitated by soil acidification, the release of phenolics/coumarins and flavins, and cation reduction when required (Jain et al., 2014). Metals are then introduced into the root epidermis and symplastically or apoplastically reach the root endodermis, to cross into the vasculature, and delivered to sink organs. In model legume *Medicago truncatula*, metals are released from the vessels into the apoplast of the infection-differentiation zone of nodules (Rodríguez-Haas et al., 2013). These nutrients will be introduced in rhizobia-infected cells and targeted to symbiosomes for nitrogen fixation. In recent years, many of the membrane transporters participating in metal transfer from the plant to the bacteroids have been identified. For instance, iron transfer to nitrogen-fixing cells is facilitated by plasma membrane iron uptake protein MtNramp1 (Tejada-Jiménez et al., 2015), and its transport across the symbiosome membrane by MtSEN1 and MtFPN2 (Hakoyama et al., 2012, Escudero et al., 2019b). However, little is known on how metals are sorted intracellularly and on the speciation of these elements.

Unlike alkali or alkali-earth elements, transition metals are not “free,” hydrated, in physiological solutions. Instead, they are bound to a plethora of organic molecules that maintain them soluble under different pH, prevent metal-catalyzed production of free radicals in Fenton-style reactions, and avoid mis-metallation of enzymes (Finney and Halloran, 2003; Rellán-Álvarez et al., 2008; Flis et al., 2016). Systematic studies of the nature of these chemical species in the sap of model plants have revealed the importance of citrate and nicotianamine in this role (von Wiren et al., 1999; Durrett et al., 2007; Roschzttardtz et al., 2011; Schuler et al., 2012). Citrate is the main iron chelator in xylem and facilitates iron delivery across symplastically disconnected tissues (Durrett et al., 2007; Rellán-Álvarez et al., 2010; Roschzttardtz et al., 2011). It has also been associated with iron trafficking to nodules (LeVier et al., 1996). Citrate efflux proteins LjMATE1 and MtMATE67 are required for iron allocation to nodules and contribute to nitrogen fixation (Takanashi et al., 2013; Kryvoruchko et al., 2018). Citrate efflux is relevant for iron delivery to bacteroids, as indicated by the symbiosome localization of nodule-specific protein MtMATE67 (Kryvoruchko et al., 2018).

Nicotianamine is also an important player in plant metal homeostasis. This molecule is a non-proteinogenic amino acid synthesized by nicotianamine synthases (NAS) from S-adenosyl methionine (Higuchi et al., 1999). Nicotianamine-metal complexes mediate long-distance metal trafficking, particularly along the phloem, as well as participate in vacuolar metal storage (von Wiren et al., 1999; Haydon et al., 2012; Flis et al., 2016). A nodule-specific NAS gene was identified in senescent nodules of *L. japonicus*, likely participating in the metal redistribution to the developing flowers and embryos, as orthologues do with older leaves (Hakoyama et al., 2009; Schuler et al., 2012). No such nodule-specific NAS gene can be found in transcriptomic databases from indeterminate type nodules, but tentative evidence shows that a *M. truncatula* NAS protein, MtNAS1, might be responsible for iron allocation to these organs (Avenhaus et al., 2016). Here, we have characterized a second NAS protein, MtNAS2, identified in a screening of *M. truncatula Tnt1*-insertion mutants. This gene, although primarily expressed in roots, is important for metal allocation for symbiotic nitrogen fixation.

## Materials and Methods

### Biological Material and Growth Conditions

*M. truncatula* Gaertn R108 and *nas2-1* (NF15101) seeds were scarified in concentrated sulfuric acid (96%) for 7.5 min. After removing the acid, the seeds were washed eight times with cold water, and surface-sterilized with 50% (v/v) bleach for 90 s. Seeds were embedded overnight in the dark at room temperature in sterile water, and transferred to 0.8% water-agar plates for 48 h at 4°C (stratification). Germination was carried out at 22°C in the dark. Seedlings were planted on sterile perlite pots, and inoculated with *Sinorhizobium meliloti* 2011 or the same bacterial strain transformed with pHC60 (Cheng and Walker, 1998). Plants were grown in a greenhouse under 16 h light/8 h dark at 25/20°C conditions. In the case of perlite pots, plants were watered every 2 days with Jenner's solution or water alternatively (Brito et al., 1994). Nodules were obtained at 28 dpi. Plants growing in non-symbiotic conditions were watered every 2 weeks with Jenner's solution supplemented with 20 mM NH_4_NO_3_. For hairy-root transformation experiments, *M. truncatula* seedlings were transformed with *Agrobacterium rhizogenes* strain ARqua1, fused to the appropriate binary vector as described (Boisson-Dernier et al., 2001). Briefly, the root tips of *M. truncatula* seedlings were removed and the wound was placed in contact with a fresh solid culture of *A. rhizogenes* ARqua1 containing the binary vector of interest. These seedlings were placed in Fahraeus medium (Fahraeus, 1957) containing 50 μg/ml kanamycin to select for transformed roots, and transferred a germination chamber at 16 h light/8 h dark at 25/20°C conditions. After 3 weeks, plants that had developed roots were placed in perlite pots and inoculated as indicated above.

### Ribonucleic Acid Extraction and Quantitative Real-Time Polymerase Chain Reaction

RNA was extracted from 28 dpi plants using TRI-reagent (Life Technologies), treated using DNase turbo (Life Technologies), and cleaned with RNeasy Mini-Kit (Qiagen). Complementary DNA (cDNA) was obtained from 500 ng RNA using PrimeScript RT reagent Kit (Takara). Expression studies were carried out by real-time reverse transcription polymerase chain reaction (RT-qPCR; StepOne plus, Applied Biosystems) using the Power SyBR Green Master Mix (Applied Biosystems). The primers used are indicated in **Supplementary Table 2**. cDNA levels were normalized by using the ubiquitin conjugating enzyme E2 (*Medtr7g116940*) gene as internal standard. Real time cycler conditions have been previously described (González-Guerrero et al., 2010). Expression levels were determined in three independent experiments with four pooled plants.

### β-Glucuronidase Staining

*MtNAS2* promoter region was obtained by amplifying the 1,940 bp upstream of the start codon using the primers indicated in **Supplementary Table 2**, and cloned by Gateway Cloning Technology (Invitrogen) in pDONR207 (Invitrogen) and transferred to destination vector pGWB3 (Nakagawa et al., 2007). Hairy-root transformations of *M. truncatula* seedlings were carried out with *A. rhizogenes* ARqua1 as described by Boisson-Dernier et al. (2001). After 3 weeks on Fahraeus media plates with kanamycin (50 µg/ml), plant transformants were transferred to sterilized perlite pots and inoculated with *S. meliloti* 2011. GUS activity was determined in 28 dpi plants as described (Vernoud et al., 1999). Whole nodule and root images were taken with a Leica MZ10F dissecting microscope using the Leica AF software. Some nodules and roots were embedded in 6% agarose and sectioned in 100 μm slides using a Vibratome 1000 Plus. These sections were observed in a Zeiss Axiophot microscope using the Leica AF software.

### Immunolocalization and Imaging

The coding sequence region of *MtNAS2* and 1,940 bp upstream of its start codon were cloned in pGWB13 vector (Nakagawa et al., 2007) using Gateway Cloning Technology (Invitrogen). This fuses three HA epitopes to C-terminus of the protein. Hairy-root *M. truncatula* transformants were transferred to sterilized perlite pots and inoculated with *S. meliloti* 2011 containing the pHC60 plasmid that constitutively expresses GFP. Nodules and roots were collected from 28 dpi plants and fixed at 4°C overnight in 4% para-formaldehyde and 2.5% sucrose in phosphate-buffered saline (PBS). Fixative was removed by washing for 5 min in PBS and 5 min in water. Nodule and roots were included in 6% agarose for sectioning with a Vibratome 1000 Plus. Sections were dehydrated by serial incubation with methanol (30, 50, 70, and 100% in PBS) for 5 min and then rehydrated following the same methanol series in reverse order. Cell wall permeabilization was carried out by incubation with 2% (w/v) cellulase in PBS for 1 h and 0.1% (v/v) Tween 20 for 15 min. Sections were blocked with 5% (w/v) bovine serum albumin in PBS and then incubated with 1:50 anti-HA mouse monoclonal antibody (Sigma) in PBS at room temperature for 2 h. Primary antibody was washed three times with PBS for 15 min and subsequently incubated with 1:40 Alexa 594-conjugated anti-mouse rabbit monoclonal antibody (Sigma) in PBS at room temperature for 1 h. Secondary antibody was washed three times with PBS for 10 min. DNA was stained using 4′,6-diamidino-2-phenylindol (DAPI). Images were obtained with a confocal laser-scanning microscope (Leica SP8) using excitation light at 488 nm to GFP and 561 nm for Alexa 594, collecting the emission at 500–554 nm for GFP and at 597-654 for Alexa594.

### Acetylene Reduction Assays

Nitrogenase activity assay was measured by acetylene reduction test (Hardy et al., 1968). Wild-type and *nas2-1* nodulated roots from 28 dpi were separately introduced in 30 ml vials. Each tube contained four or five independently transformed plants. Three milliliters of air from each bottle was replaced by the same volume of acetylene, tubes were subsequently incubated for 30 min at room temperature. Gas samples were measured by analyzing 0.5 ml of ethylene from each bottle in a Shimadzu GC-8A gas chromatograph using a Porapak N column. The amount of the ethylene produced was determined by measuring the ethylene peaks relative to the standards. Acetylene reduction was measured in duplicate from three sets of three-four pooled plants.

### Chlorophyll Content Assays

Total chlorophyll content was determined as previously described with some modifications (Inskeep and Bloom, 1985). Leaves were collected from 28 dpi plants and poled to obtain 50 mg of fresh material. Chlorophyll was extracted with 500 µl of di-methyl-formamide at 4°C overnight. Leaves were centrifuged for 5 min at 600 g at room temperature. After transferring the supernatant to another vial, the chlorophyll extraction was repeated with the same leave using strong vortexing. After spinning for 5 min at 600 g, the supernatant was pooled with the previous one. Chlorophyll was quantified at 647 and 664 nm in a Ultrospec 3300 Spectrophotometer (Amersham Bioscience). Data are the mean ± SE of three sets of five pooled plants.

### Metal Content Determination

Shoots, roots, and nodules were collected from 28 dpi plants and mineralized with 15.6 M HNO_3_ (trace metal grade) at 75°C for 3 h and 2 M H_2_O_2_ at 20°C overnight. Metal quantifications were performed in duplicate by Atomic Absorption Spectroscopy, using a Perkin Elmer PinAAcle 900Z GF-AAS equipment. Metal concentration was normalized against fresh tissue weight. Three sets of three-four pooled organs were analyzed.

### Synchrotron Radiation X-Ray Fluorescence Spectroscopy and X-Ray Absorption Near-Edge Spectroscopy

X-ray fluorescence spectroscopy (XRF) hyperspectral images and µXANES spectra were acquired on the beamline ID21 of the European Synchrotron Radiation Facility (Cotte et al., 2017), at 110 K in the liquid nitrogen (LN2) cooled cryostat of the Scanning X-ray Micro-spectroscopy end-station. Seven sections from *M. truncatula* R108 nodules and five from *nas2-1* nodules were obtained from independent nodules embedded in optimal cutting temperature (OCT) medium and cryo-fixed by plunging in isopentane chilled with LN2. The 25 µm-thick sections of frozen samples were obtained using a Leica LN2 cryo-microtome and accommodated in a Cu sample holder cooled with LN2, sandwiched between Ultralene (SPEX SamplePrep) foils. The beam was focused to 0.4×0.9 µm^2^ with a Kirkpatrick-Baez (KB) mirror system. The emitted fluorescence signal was detected with energy-dispersive, large area (80 mm^2^) SDD detector equipped with Be window (SGX from RaySpec). Images were acquired at the fixed energy of 7.2 keV, by raster-scanning the sample in the X-ray focal plane, with a step of 3×3 µm^2^ and 100 ms dwell time. Elemental mass fractions were calculated from fundamental parameters with the PyMCA software package, applying pixel-by-pixel spectral deconvolution to hyperspectral maps normalized by the incoming flux (Solé et al., 2007). The incoming flux was monitored using a drilled photodiode previously calibrated by varying the photon flux at 7.2 keV obtaining a response of 1,927.9 charges/photon with a linear response up to 200 kcps. In PyMCA the incoming flux and XRF detector parameters were set to 2x10^9^ photons/s, 0.7746 cm^2^ active area, and 4.65 cm sample to XRF detector distance. Sample matrix was assumed to be amorphous ice (11% H, 89% O, density 0.92 g/cm^3^), the sample thickness set at 25 µm obtained with the use of a cryo-microtome. Iron average mass fraction values from pixels in zone III and apical region from three wild type and three *nas2-1* nodule samples were obtained using a mask tool that removes pixels with mass fractions below a threshold of 2 10^−6^ μg iron.

Fe-K edge (7.050 to 7.165 keV energy range, 0.5 eV step) µXANES spectra were recorded in regions of interest of the fluorescence maps acquired on ID21 beamline. Individual spectra were processed using Orange software with the Spectroscopy add-on (Demsar et al., 2013). The pre-processing step consisted of vector normalization and a Savitzky-Golay filter for the smoothing. Then a principal component analysis was performed on the second derivative of the spectra to highlight potential differences among genotypes within a given region of the nodule. A reference library was used for linear combination fitting (LCF) procedure. This library consisted of: Fe-foil [Fe(0)], Fe(II)-nicotianamine, Fe(II)S2 (http://ixs.iit.edu/database/), Fe(III)-haem (50 mM, pH7, bought from Sigma, CAS number: 16009-13-5), Fe(III)-cellulose (5 mM FeCl_3_ + 50 mM cellulose, pH 5.8, bought from Sigma, CAS number: 9004-34-6), Fe(III) glutamic acid (5 mM FeCl_3_ + 50 mM glutamic acid, pH 7, bought from Sigma, CAS number: 56-86-0), and Fe(III) ferritin (bought from Sigma, CAS number: 9007-73-2, 50 mM, pH7).Reference compounds were classified as Fe(II)-S (FeS2), Fe(II)-O/N (Fe-NA) and Fe(III)-O (Fe-cellulose, Fe-glutamic acid, and ferritin). XANES data treatment was performed using Athena software (Ravel and Newville, 2005) as previously described (Larue et al., 2014).

### Statistical Tests

GraphPad Prism was used for the statistical analyses. Data were analyzed by ANOVA to test the differences between wild type, mutant and complemented *M. truncatula* plants. Tukey's or Student's *t* tests were used to calculate statistical significance of observed differences. Test results with *p*-values < 0.05 were considered as statistically significant

For nodule structure analysis and nicotianamine concentration determination see **Supplementary Materials** and Methods.

## Results

### *Medicago truncatula Tnt1* Line NF15101 Phenotype Is Due to Transposon Insertion in *MtNAS2*

**Figure 1:**
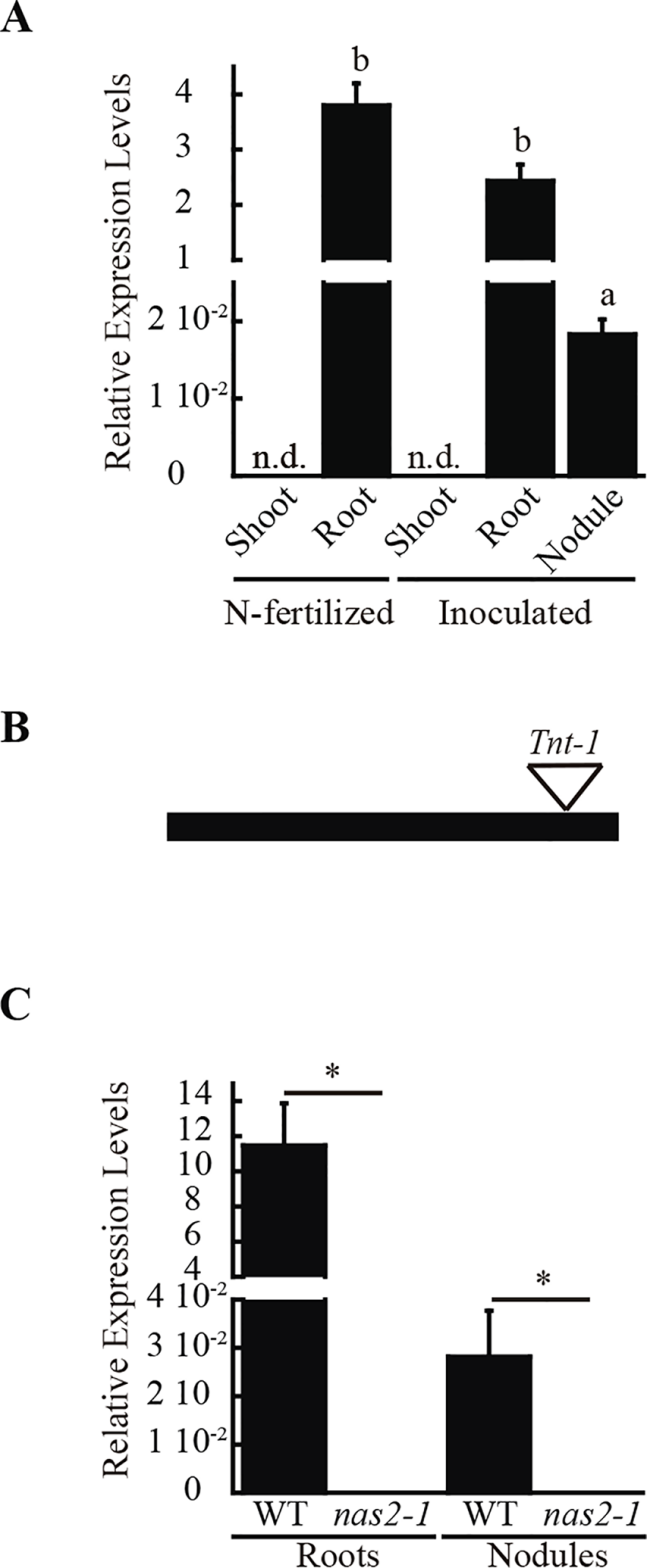
*MtNAS2* is expressed in roots and nodules of *M. truncatula*. **(A)** Expression data was normalized to the expression of ubiquitin conjugating enzyme E2 gene (*Medtr7g116940*) as standard. Data are the mean ± SE of three independent experiments with 4 pooled plants. Different letters indicate statistical differences at P > 0.05 (Tukey t-test). **(B)** *Tnt1* insertion in the only exon of MtNAS2 causes loss of *MtNAS2* transcripts. **(C)** MtNAS2 expression was determined in 28 dpi roots and nodules of wild-type (WT) and nas2-1 plants. Data was relativized to the expression of ubiquitin conjugating enzyme E2 (*Medtr7g116940*) and expressed as mean ± SE of three independent experiments with 4 pooled plants. *indicates statistical differences at P > 0.05 (Student t-test).

**Figure 2:**
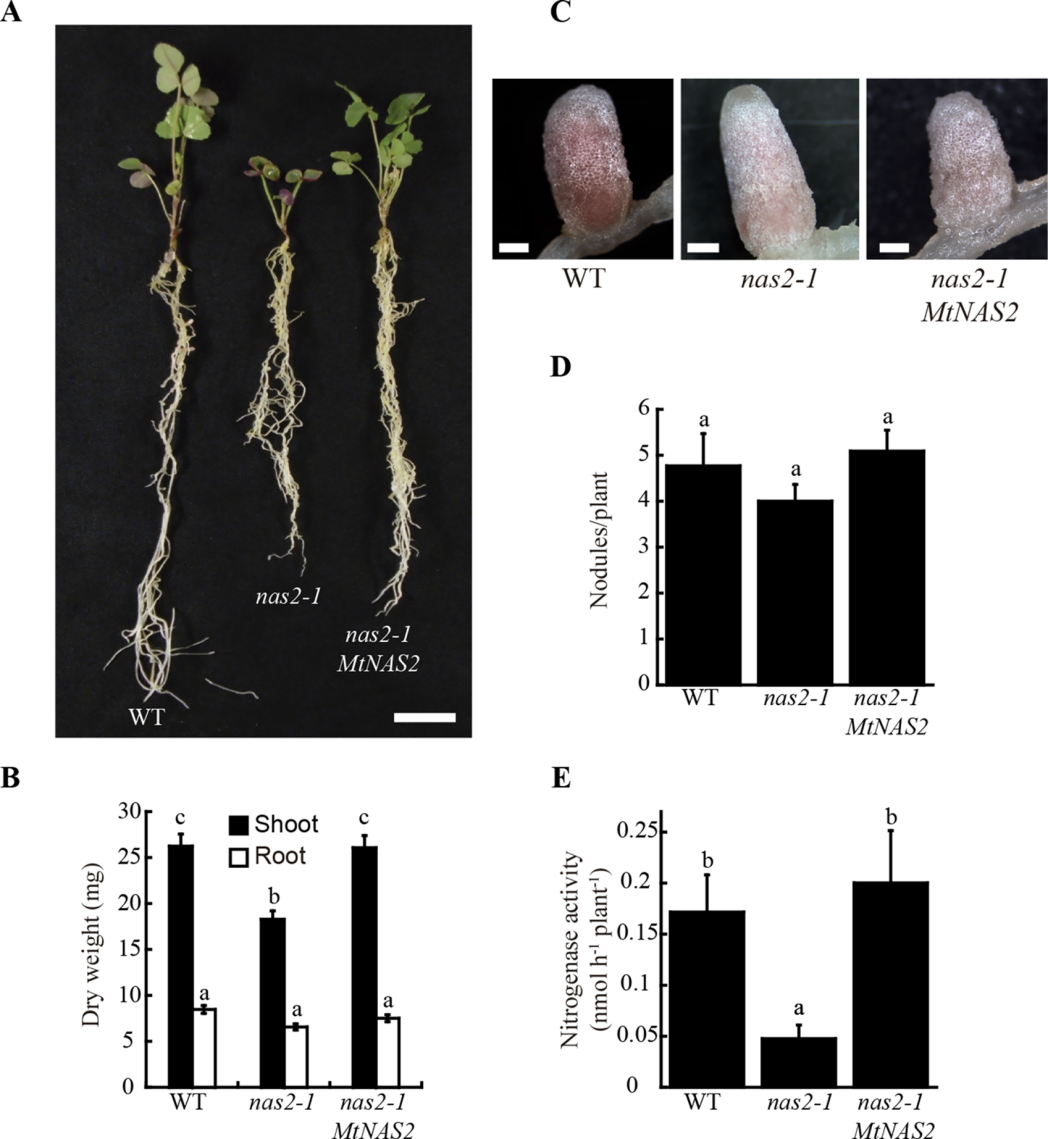
*MtNAS2* is required for nitrogen fixation. **(A)** Growth of representative wild-type (WT), *nas2-1*, and *nas2-1* plants transformed with *MtNAS2* controlled by its own promoter (*nas2-1 MtNAS2*). Bar = 1.5 cm. **(B)** Dry weight of WT, *nas2-1*, and *nas2-1 MtNAS2* plants. Data are the mean ± SE of at least 9 transformed plants. **(C)** Detail of representative nodules of WT, *nas2-1*, and *nas2-1 MtNAS2* plants (n = 40–50 nodules). Bars = 500 μm. **(D)** Number of nodules in 28 dpi WT, *nas2-1*, and *nas2-1 MtNAS2* plants. Data are the mean ± SE of at least nine transformed plants. Different letters indicate statistical differences at P < 0.05 (Tukey t-test). **(E)** Nitrogenase activity in 28 dpi nodules from WT, *nas2-1*, and *nas2-1 MtNAS2* plants. Acetylene reduction was measured in duplicate from three sets of three-four pooled plants. Data are the mean ± SE. Different letters indicate statistical differences at P < 0.05 (Tukey t-test).

A search for metal-related symbiotic phenotypes of the mutants available at the Noble Research Institute LLC *M. truncatula* Mutant Database (Sun et al., 2019; https://medicago-mutant.noble.org/mutant/index.php) showed NF15101 as one of the available mutants with a nitrogen fixation deficient phenotype. This line has 22 *Tnt1* insertions, 10 of which interrupted different *M. truncatula* genes (**Supplementary Table 1**), *Medtr2g070310* among them. This gene encodes a protein with 52% identity and 67% similarity to *Arabidopsis thaliana* NAS2 protein, and consequently was renamed *MtNAS2*. *MtNAS2* was expressed at similar levels in roots from plants inoculated or non-inoculated with *S. meliloti* (**Figure 1A**). Significantly, lower expression was observed in nodules, and no signal was detected in shoots from either inoculated or non-inoculated plants. *Tnt1* was inserted in position +760 of *MtNAS2* (**Figure 1B**), interrupting the reading frame of its only exon. Homozygous plants from a R2 seed population were identified by a PCR reverse screening (Cheng et al., 2014). These homozygous plants were considered as null mutants for *MtNAS2*, since *MtNAS2* expression levels were below our detection limit (**Figure 1C**). As expected, NF15101, *nas2-1* in this report, had reduced biomass production in nitrogen fixation conditions (**Figures 2A, B**). While nodule development and nodule number were not significantly altered in *nas2-1* compared to wild type (**Figures 2C, D**, **Supplementary Figure 1**), nitrogenase activity was reduced three-fold in *nas2-1* plants (**Figure 2E**). No significant differences in nicotianamine concentration were observed between wild-type and mutant plants (**Supplementary Figure 2**). The *nas2-1* phenotype was reverted when a wild-type copy of *MtNAS2* regulated by its own promoter was reintroduced in *nas2-1* (**Figure 2**). No significant changes in nitrogenase activity were observed either when iron and/or zinc were removed from the nutrient solution or when they were added at higher concentrations (**Supplementary Figure 3**) The data indicate that among all the *Tnt1* insertions, loss of *MtNAS2* function was determinant for the reduction of nitrogenase activity and overall growth alterations.

### *MtNAS2* Is Not Required for Plant Growth Under Non-Symbiotic Conditions

**Figure 3:**
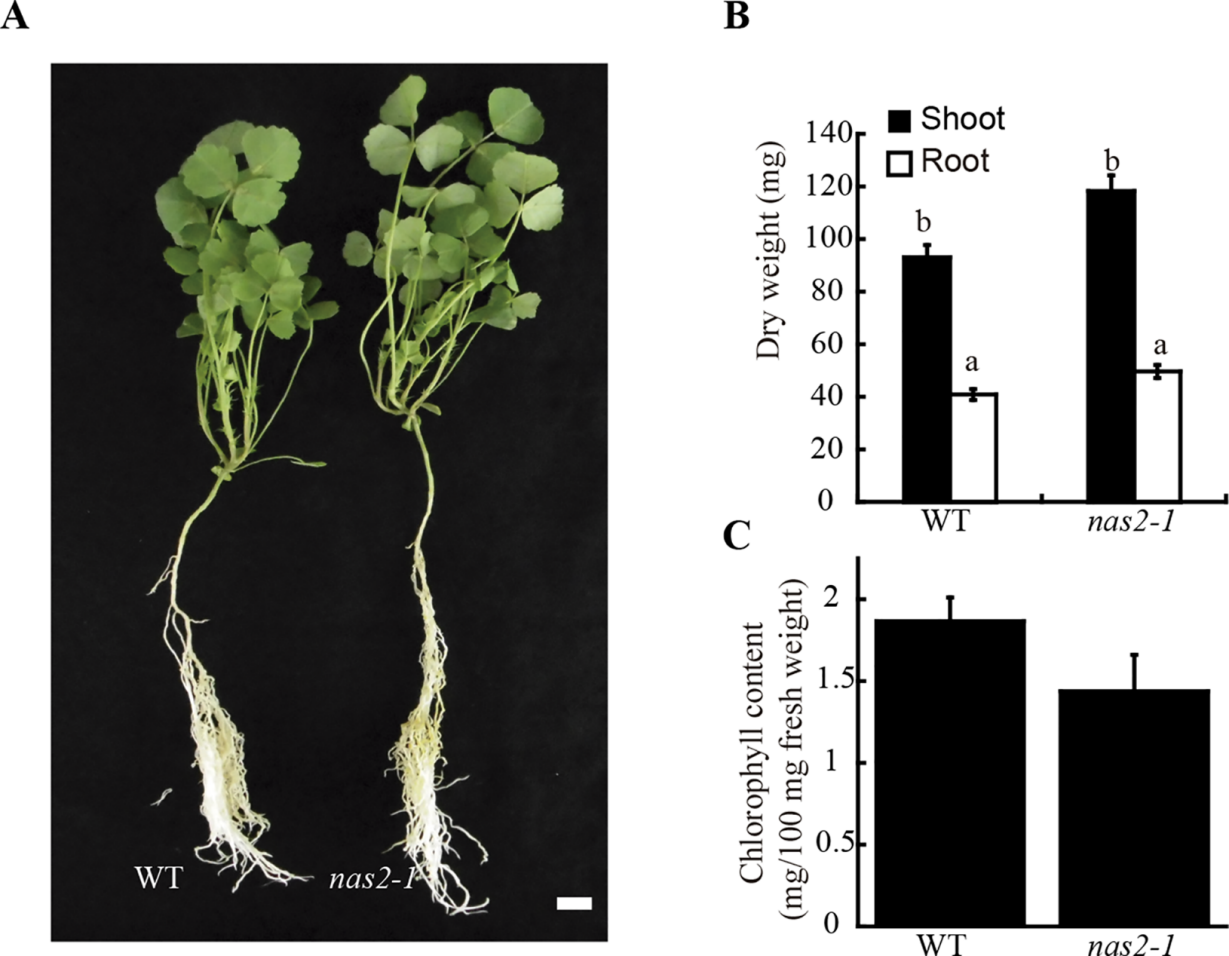
*MtNAS2* is not required for plant growth under non-symbiotic conditions. **(A)** Growth of representative wild-type (WT) and *nas2-1* plants when watered with a nutrient solution supplemented with ammonium nitrate and not inoculated with *Sinorhizobium meliloti* (n = 10–15 plants). Bar = 1.5 cm. **(B)** Dry weight of WT and *nas2-1* plants. Data are the mean ± SE (n = 10 plants). Different letters indicate statistical differences at P < 0.05 (Tukey t-test). **(C)** Chlorophyll concentration of wild-type and *nas2-1* plants. Data are the mean ± SE of three sets of five pooled plants. No differences at P < 0.05 were observed (Student t-test).

To determine whether the symbiotic phenotype of *nas2-1* was the result of additional physiological processes being affected, these plants and their controls were grown in the same conditions as above, but supplemented with ammonium nitrate in the nutrient solution to compensate for the lack of rhizobial inoculation. In these conditions, no significant differences were found in plant growth, biomass production, or chlorophyll content between wild-type and *nas2-1* plants (**Figure 3**). Considering the role of nicotianamine in plant iron homeostasis (von Wiren et al., 1999; Inoue et al., 2003) and the added pressure of symbiotic nitrogen fixation on iron nutrition (Terry et al., 1991), *nas2-1* phenotype was also studied under non-symbiotic, low-iron conditions (no iron added to the nutrient solution). Low-iron supply did not lead to different growth between control and *nas2-1* plants (**Supplementary Figure 4**).

### *MtNAS2* Is Expressed in the Xylem Parenchyma in Roots and in the Nodule Differentiation and Fixation Zones

**Figure 4:**
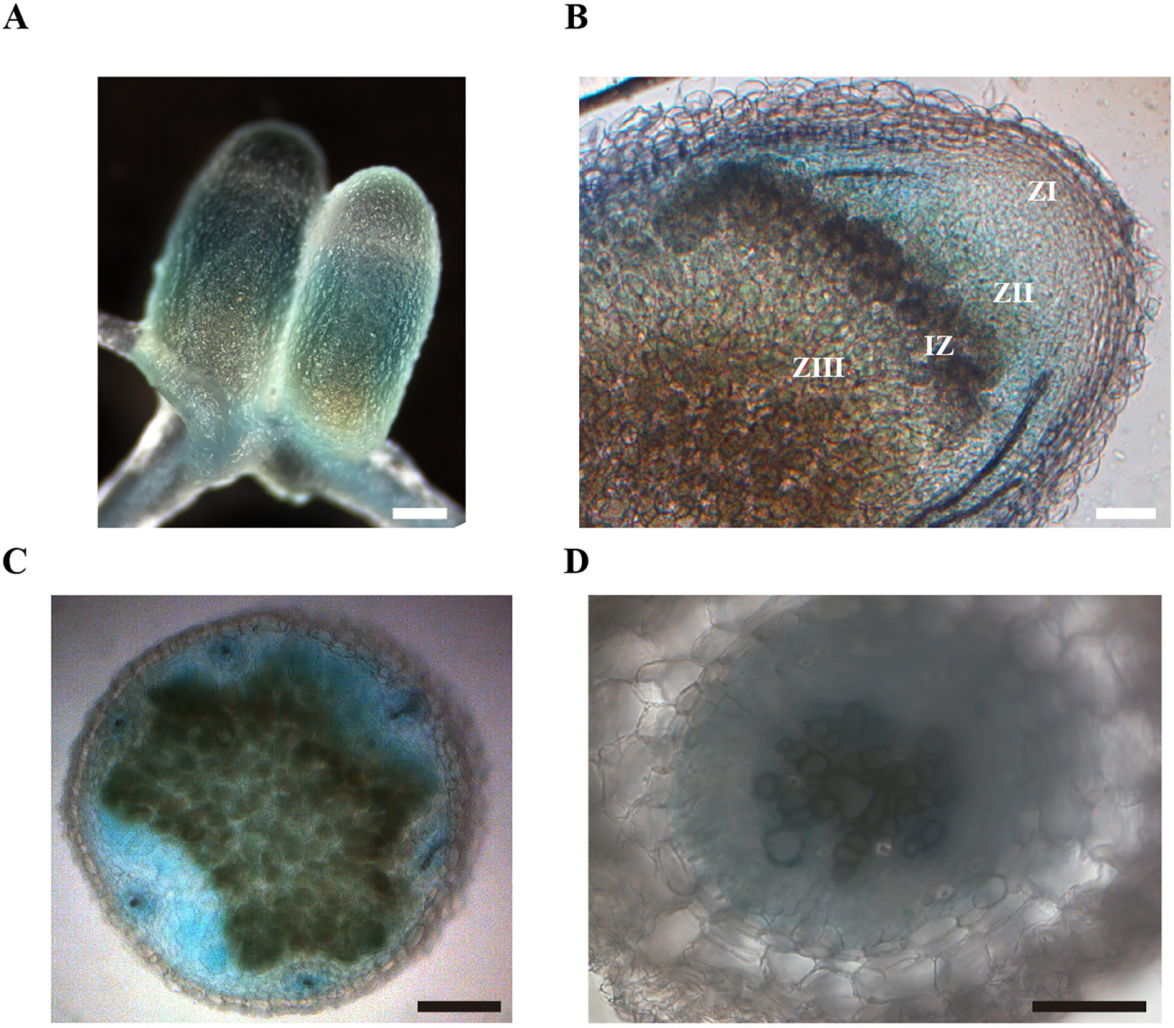
*MtNAS2* is expressed in the root vasculature and in the late zone II, interzone, zone III, and vessels in nodules. **(A)** β-glucuronidase (GUS) staining of 28 dpi *Medicago truncatula* roots and nodules expressing the *gus* gene under the control of *MtNAS2* promoter region (n = 10–20 plants). Bar = 100 μm. **(B)** Longitudinal section of a GUS-stained 28 dpi *M. truncatula* nodule expressing the *gus* gene under the control of *MtNAS2* promoter region (n = 30 sections from 10 to 20 plants). ZI indicates zone I; ZII, zone II; IZ, interzone; and ZIII, zone III. Bar = 100 μm. **(C)** Cross section of the fixation zone of a GUS-stained 28 dpi *M. truncatula* nodule expressing the *gus* gene under the control of *MtNAS2* promoter region (n = 30 sections from 10 to 20 plants). Bar = 200 μm. **(D)** Cross section of a GUS-stained 28 dpi *M. truncatula* root expressing the *gus* gene under the control of *MtNAS2* promoter region (n = 30 sections from 10 to 20 plants). Bar = 100 μm.

The physiological role of *MtNAS2* is determined by its differential tissue and cellular expression. To establish the gene tissue expression, *M. truncatula* plants were transformed with a binary vector containing the *MtNAS2* promoter region driving the *β-glucuronidase* (*gus*) gene transcription and GUS activity visualized using X-Gluc. *MtNAS2* was expressed in roots and nodules (**Figure 4A**), in agreement with the transcript data (**Figure 1**). Longitudinal section of the nodules showed GUS activity in cells from the late zone II to the fixation zone of the nodule (**Figure 4B**). Nodule cross-sections showed expression in all nodule tissues (**Figure 4C**). In roots, *MtNAS2* promoter was active in vasculature cells (**Figure 4D**).

**Figure 5:**
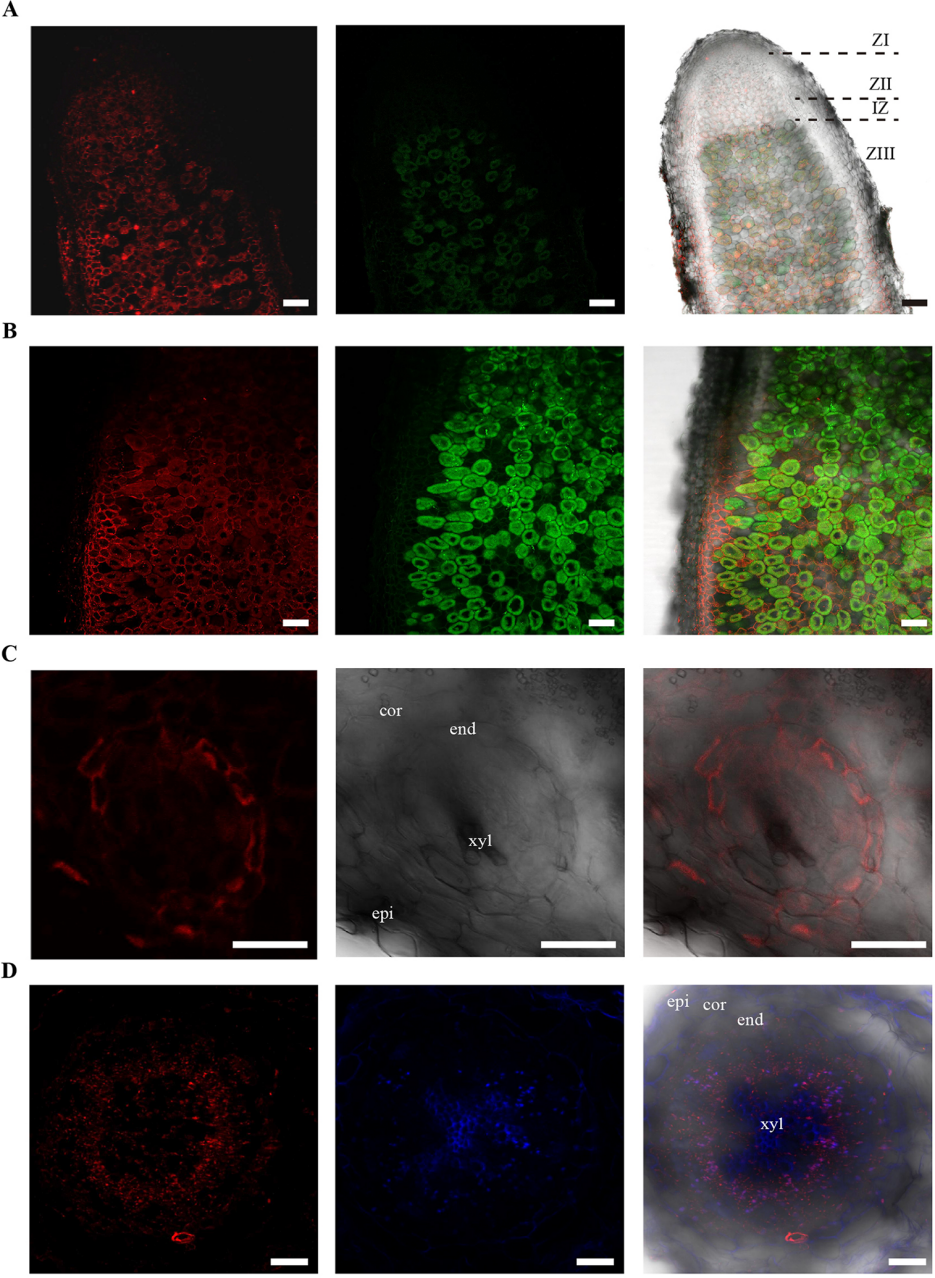
MtNAS2 is located in the nodule core cells, in the endodermis of the nodule vessels, and in cells surrounding the xylem in the root vasculature. **(A)** Longitudinal section of a 28 dpi *M. truncatula* nodule expressing *MtNAS2-HA* under its own promoter (n = 30 sections from 10 to 20 plants). The three C-terminal HA epitopes were detected using an Alexa594-conjugated antibody (red, left panel). Transformed plants were inoculated with a GFP-expressing *Sinorhizobium meliloti* (green, middle panel). Both images were overlaid with the transillumination image (right panel). ZI indicates zone I; ZII, zone II; IZ, interzone; and ZIII, zone III. Bars = 100 μm. **(B)** Detail of the zone III of a 28 dpi *M. truncatula* nodule expressing *MtNAS2-HA* under its own promoter. Left panel corresponds to the Alexa594 signal used to detect the HA-tag, middle panel corresponds to the GFP channel showing *S. meliloti*, and the two were overlaid with the bright field channel in the right panel (n = 30 sections from 10 to 20 plants). Bars = 50 μm **(C)** Cross section of a nodule vessel from a 28 dpi *M. truncatula* nodule expressing *MtNAS2-HA* under its own promoter. Left panel corresponds to the Alexa594 signal used to detect the HA-tag, middle panel corresponds to the bright field channel, and the two were overlaid in the right panel (n = 30 sections from 10 to 20 plants). epi indicates nodule epidermis; cor, nodule cortex; end, nodule vascular endodermis; and xyl, nodule vascular xylem. Bars = 50 μm. **(D)** Cross section from a 28 dpi *M. truncatula* root expressing *MtNAS2-HA* under its own promoter. Left panel corresponds to the Alexa594 signal used to detect the HA-tag, middle panel corresponds to autofluorescence signal of xylem, and the two were overlaid with the bright field channel in the right panel (n = 30 sections from 10 to 20 plants). epi indicates root epidermis; cor, root cortex; end, root endodermis; and xyl, xylem. Bars = 100 μm.

Supporting the gene expression results, immunolocalization of HA-tagged MtNAS2 under control of its own promoter showed that the protein was located in cells neighboring the late zone II, interzone, and fixation zone (**Figure 5A**). At higher magnification, we could observe that MtNAS2-HA had a homogenous distribution within the cells, and it did not seem to cluster in any particular location (**Figure 5B**). Analysis of nodule vasculature showed MtNAS2-HA in endodermal cells (**Figure 5C**). However, in the root vasculature, MtNAS2-HA was detected at the center of the root, in the region comprised between the endodermis and the xylem (**Figure 5D**). At higher magnification, the root MtNAS2-HA signal was detected in small cells associated to the xylem (**Supplementary Figure 5**). Controls were carried out to ensure that the data did not stem from autofluorescence (**Supplementary Figure 6**). In addition, the antibodies used for immunolocalization do not cross react with *M. truncatula* proteins (Tejada-Jiménez et al., 2015).

### MtNAS2 Is Required for Efficient Metal Allocation for Symbiotic Nitrogen Fixation

**Figure 6:**
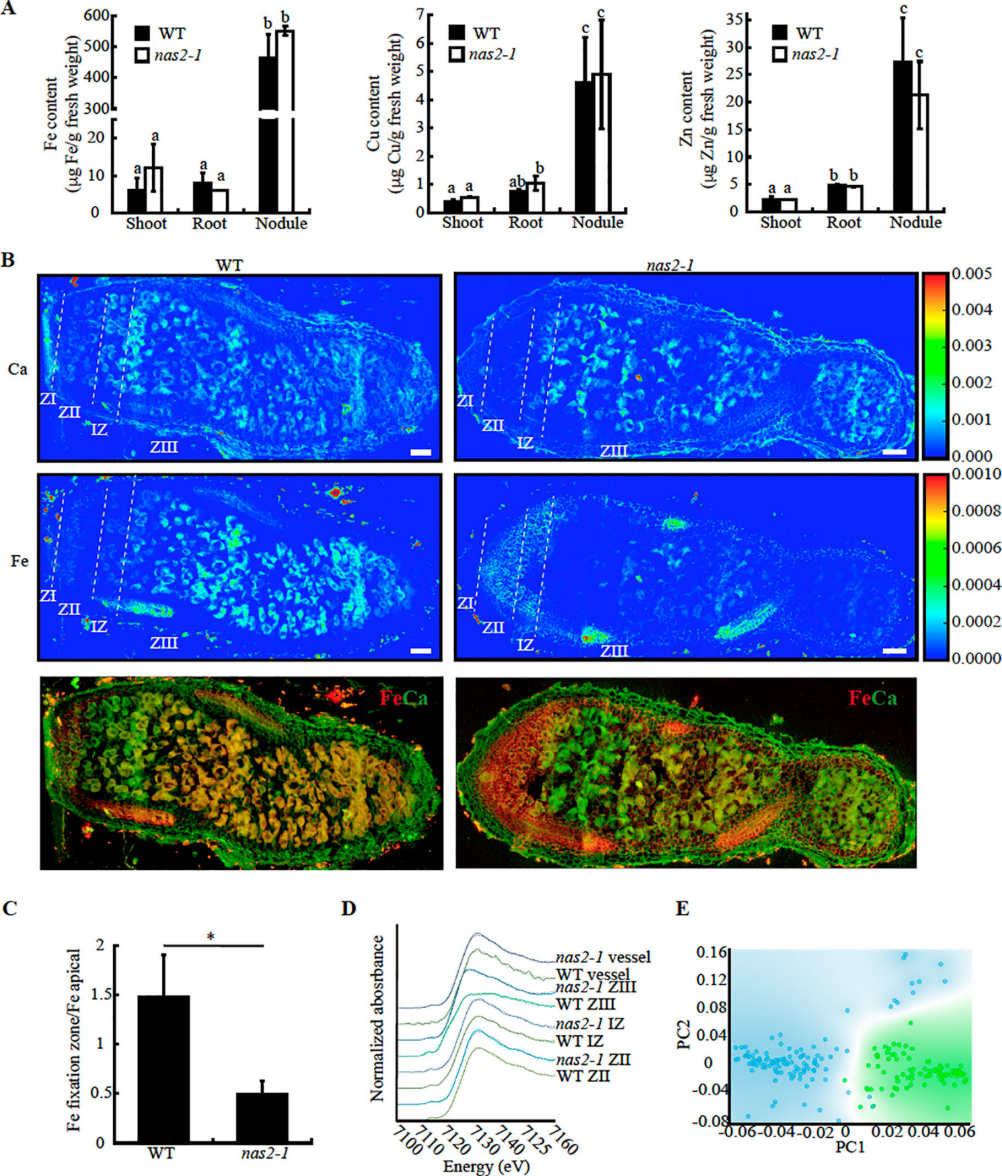
*MtNAS2* is required for iron distribution and speciation in nodules. **(A)** Iron (left panel), copper (middle panel), and zinc (right panel) concentration in shoots, roots, and nodules from 28 dpi wild-type (WT) and *nas2-1* plants. Data are the mean ± SE of three sets of three-four pooled organs. Different letters indicate statistical differences at P < 0.05 (Tukey t-test). **(B)** Synchrotron-based X-ray fluorescence images of WT (left panels) or *nas2-1* (right panels) showing calcium (top panels) or iron (center panels) distribution in 28 dpi nodules. Lower panels are the overlaid iron and calcium distribution (iron is indicated in red and calcium in green). ZI indicates zone I; ZII, zone II; IZ, interzone; and ZIII, zone III (n = 5–7 nodules). Bars = 100 μm. **(C)** Iron content in fixation zone relative to the apical zone in wild type and *nas2-1* nodules. Data are the mean ± SE (n = 3). * indicates statistical differences at P < 0.05 (Student t-test). **(D)** XANES spectra obtained from different regions of WT and *nas2-1* nodules. **(E)** Decomposition of the zone III signal into its two principal components.

Nicotianamine is required for metal allocation from source to sink tissues (Schuler et al., 2012). Alterations in nicotianamine synthesis typically lead to reduced metal delivery to sink tissues. To determine whether this was the case for *nas2-1*, iron, copper, and zinc levels in roots, shoots, and nodules from 28 days-post-inoculation (dpi) plants were determined. No significant changes in these levels were observed (**Figure 6A**). However, metal allocation might be altered while not affecting total nodule metal content. To assess this possibility, synchrotron-based X-ray fluorescence studies were carried out to determine iron distribution in *nas2-1* compared to wild type (**Figure 6B**). These experiments showed that iron distribution was altered in *nas2-1* mutants, which presented a lower percentage of iron in the fixation zone than wild type nodules (**Figure 6C**). To further confirm that mutation of *MtNAS2* affected iron distribution in nodules as a consequence of changes of iron speciation, X-ray Absorption Near-Edge Spectroscopy (XANES) analyses of iron speciation in the different nodule developmental zones were carried out (**Figure 6D**). Principal component analyses of these spectra showed that the iron complexes in the fixation zone were quite different (**Figure 6E**). Fitting of the obtained spectra to known standards showed that the proportion of Fe-S complexes had a dramatic drop in *nas2-1* compared to wild-type plants, while the proportion of O/N complexes with iron had a larger increase (**Table 1**).

**Table 1:** Iron speciation (%) in wild type (WT) and *nas2-1* nodules. n.d., not detected.

Region	WT				*nas2-1*			
	Fe(II)-S	Fe(II)-O/N	Fe(III)-O	R^2^	Fe(II)-S	Fe(II)-O/N	Fe(III)-O	R^2^
ZII	4	12	83	00004	n.d.	14	86	0.0008
IZ	13	19	69	0.0004	5	17	78	0.0006
ZIII	50	24	27	0.001	14	49	37	0.0005
Vessels	n.d.	22	79	0.0008	n.d.	22	78	0.001

## Discussion

Metallic micronutrient delivery to nodules is essential for symbiotic nitrogen fixation, as they are cofactors in many of the involved enzymes (Brear et al., 2013; González-Guerrero et al., 2014). In recent years, studies have shown how metals are exported to the apoplast in the infection/differentiation zone of *M. truncatula* nodules (Rodríguez-Haas et al., 2013), and transmembrane transporters introduce metals into rhizobia-infected cells (Tejada-Jiménez et al., 2015; Abreu et al., 2017; Tejada-Jiménez et al., 2017; Senovilla et al., 2018), or deliver iron to the bacteroids (Escudero et al., 2019b). In this transport, citrate participates in maintaining iron solubility in the apoplast, and as the preferred iron source for bacteroids (Moreau et al., 1995; LeVier et al., 1996; Kryvoruchko et al., 2018). Here we show that nicotianamine synthesis is also important for correct iron allocation to *M. truncatula* nodules.

Interrupting *MtNAS2* expression with a transposon insertion led to reduced plant growth in symbiotic conditions, a consequence of lower nitrogenase activity. Although several genes were affected in the studied *Tnt1* line, reintroduction of a wild-type copy of *MtNAS2* was sufficient to restore wild-type growth. Consequently, the mutation of this gene was mainly responsible for the observed phenotype. While important under symbiotic conditions, *MtNAS2* seemed to be playing a secondary role when plants were not inoculated but watered with an ammonium nitrate-supplemented nutrient solution instead. This is in contrast to the substantially higher expression levels of *MtNAS2* in roots than in nodules. This observation would suggest a predominant role in nicotianamine synthesis in roots. However, studies in *A. thaliana* reveal the existence of a high redundancy rate in the NAS family, where a quadruple *nas* mutant was required to observe a substantial phenotype, including limited growth (Klatte et al., 2009). Similarly, no significant changes in nicotianamine content were observed in single *nas A. thaliana* lines, as neither was observed in *M. truncatula nas2-1*. Two possible causes might explain the symbiosis-specific phenotype of *nas2-1* plants. One of them is that MtNAS2 would be required to compensate for the enhanced iron requirements of nodulated plants. This additional nutritional pressure would trigger the observed *nas2-1* phenotype. If so, we should have also observed a similar phenotype when plants were watered with an iron-restricted nutrient solution, which has been shown in the past to elicit the iron deficiency response in *M. truncatula* (Andaluz et al., 2009; Tejada-Jiménez et al., 2015). However, this was not observed. Alternatively, in a more parsimonious mechanism, neofunctionalization of pre-existing genes during the development of symbiotic nitrogen fixation might have led to the loss of functional redundancy. Similar observations have been made when studying other *M. truncatula* metal homeostasis genes that, although expressed in roots and in nodules, exhibit phenotypes limited to nodulation and nitrogen fixation (Tejada-Jiménez et al., 2015; Abreu et al., 2017; León-Mediavilla et al., 2018).

In roots, *MtNAS2* was expressed at high levels in xylem parenchyma cells, similarly to rice *NAS2* (Inoue et al., 2003). Vascular localization of NAS proteins is not unusual, since they have been associated to long distance metal trafficking (von Wiren et al., 1999; Kumar et al., 2017). In nodules, MtNAS2 was also associated to the vessels. However, the cellular localization of the protein is different to what was observed in roots; in nodules, most of vascular MtNAS2 was confined to the endodermis. This alternative distribution of MtNAS2 in vessels could be indicative of differential functions. Root vascular localization could indicate a role in metal loading of the vascular fluids, while endodermal localization in nodules might mediate either uptake from saps or intracellular metal trafficking. In any case, it seems unlikely that the nicotianamine synthesized by nodule endodermal cells would end up in the apoplast, since citrate-iron complexes seem to be formed in this compartment at a pH that does not facilitate iron-nicotianamine association (Rellán-Álvarez et al., 2008).

*MtNAS2* expression in nodule core cells in the late zone II, interzone, and zone III also indicates a role of nicotianamine in metal homeostasis of nitrogen fixing cells. It has been previously described that nicotianamine can participate in intracellular metal trafficking and in cell-to-cell metal delivery, as well as serve as intracellular storage of metals (Haydon et al., 2012). Mutation of *MtNAS2* did not significantly alter iron, copper, or zinc levels in any of the plant organs analyzed, but a major shift in iron distribution was observed in nodules, with a significant decrease of iron accumulation in the interzone and early fixation zone. This would indicate that iron trafficking in these cells is altered. However, MtNAS2-mediated iron trafficking would only affect a subset of the nodule iron-proteome, since delivery to the fixation zone was not completely blocked as attested by the red color of nodules, indicative that leghemoglobin (an important iron sink) was being produced in addition to a residual nitrogenase activity. This could suggest the existence of differential metalation pathways in nodules that might serve different subsets of proteins, which could partially complement each other under stress conditions. Supporting this hypothesis, mutation of *MtNAS2* did not equally affect all the iron species in the fixation zone. While the percentage of iron-sulfur complexes detected by XANES was significantly lower than in control plants, iron coordinated by nitrogen or oxygen atoms was increased. Considering the high demand for iron-sulfur clusters for nitrogenase assembly (Rubio and Ludden, 2005), its decrease could explain the reduction of nitrogenase activity observed. The changes in iron speciation were particularly severe in the fixation zone, which is consistent with *MtNAS2* distribution, with the observed reduction of nitrogenase activity, and with the iron distribution data. It is important to indicate that we cannot rule out similar effects on copper or zinc speciation and distribution, since the synchrotron setup available to us at the European Synchrotron Radiation Facility prevented us to carry out similar analyses on those two elements.

This work highlights the importance of MtNAS2 in iron delivery for symbiotic nitrogen fixation. This is not the only NAS gene that might be involved in the process, since total nicotianamine production is sustained in nodules, and other family members have been shown to be expressed in these organs, such as *MtNAS1* (Avenhaus et al., 2016) or *MtNAS3* (*Medtr7g112130*) according to the Symbimics database (Roux et al., 2014). However, no *MtNAS4* (*Medtr2g034240*) expression in roots or nodules is reported in this database. The localization of MtNAS2 indicates that nicotianamine would be involved in intracellular iron trafficking that is highly important for nitrogenase functioning. This role would not be directly providing the element to the bacteroid, since iron-citrate seems to be the key here, but perhaps would shuttle this element in the cytosol. However, to better define this possibility, new tools in elemental imaging and speciation with higher resolution within a cell need to be established to track iron and other elements. In addition, the roles of zinc-induced facilitator (ZIF)-like (Haydon et al., 2012) and yellow stripe-like (YSL) proteins (Waters et al., 2006) in symbiotic nitrogen fixation must be determined. Finally, other NAS proteins might facilitate iron recycling in *M. truncatula* nodules, as it occurs in *L. japonicus* (Hakoyama et al., 2009).

## Data Availability Statement

All datasets generated for this study are included in the article/**Supplementary Material**.

## Author Contributions

VE carried out most of the experimental work. IA, CL, and HC-M carried out the synchrotron-based work. IA, JC-G, JA, and AÁ-F were responsible for the nicotianamine content determination. ES performed the phenotypic analyses under changing metal concentrations and participated in the complementation assays. MT-J carried out the initial expression analyses. JW and KM obtained the *nas2-1* mutant. LN-A and JMA determined metal concentrations. JI and MG-G were responsible for experimental design, data analyses, and wrote the manuscript with contributions from all authors.

## Funding

This research was funded by a European Research Council Starting Grant (ERC-2013-StG-335284) and a Ministerio de Economía y Competitividad (MINECO) grant (AGL2015-65866-P), to MG-G, and a MINECO grant (AGL2016-75226-R) to JA and AÁ-F. VE was partially funded by the Severo Ochoa Programme for Centres of Excellence in R&D from Agencia Estatal de Investigación of Spain (grant SEV-2016-0672) to CBGP. IA is recipient of a Juan de la Cierva- Formación postodoctoral fellowship from Ministerio de Ciencia, Innovación y Universidades (FJCI-2017-33222). Development of *M. truncatula Tnt1* mutant population was, in part, funded by the National Science Foundation, USA (DBI-0703285) to KM.

## Conflict of Interest

The authors declare that the research was conducted in the absence of any commercial or financial relationships that could be construed as a potential conflict of interest.
