# Supplementary material for: Nicotianamine Synthase 2 Is Required for Symbiotic Nitrogen Fixation in Medicago truncatula Nodules
Source: Front Plant Sci. 2020 Jan 30;10:1780. doi: 10.3389/fpls.2019.01780 (PMC7003136; doi:10.3389/fpls.2019.01780)
Supplement: Supplementary file 1 [file DataSheet_1.pdf]

Supplementary Material

1 Supplementary Materials and Methods

1.1 Nodule structure analyses

Sections for nodule structural organization were obtained from 28 days-post-inoculation (dpi) nodules fixed in 1 % formaldehyde, 0.5 % glutaraldehyde, 2.5% sucrose in 50 mM potassium phosphate buffer (pH 7.4) at 4 °C for 14 – 16 h in gentle agitation. Samples were dehydrated with ethanol series and embedded in LR-White resin (London Resin Company Ltd, UK). Nodule were placed in gelatine capsules, filled with LR-White resin and polymerized at 60 °C for 24 h. Serial thin section of 1 µm, were cut using a diamond knife in a Reichert Ultracut S-Ultramicrotrome (Leyca), stained with a mixture of 1 % toluidine blue in aqueous 1 % di-sodium tetra-borate in water Direct observation of sections was perfected by a Zeiss Axiophot photomicroscope (Carl Zeiss, Oberkochen, Germany) with pictures taken with a digital camera (Leica DFC 420C, Leica).

1.2 Nicotianamine concentration

Nicotianamine was extracted as previously described (Banakar et al., 2017) with some modifications. Briefly, nicotianamine was extracted from approximately 200 mg of roots and shoots or from 50 mg of nodules, and frozen grinded in 400 µl miliQ water spiked with nicotyl-lysine (9 µM final concentration) as internal standard. Samples were homogenized in a mixer mill (Retsch MM300, Retsch) during 5 minutes at 30^{-1} sec frequency, and then centrifuged at 15000 g for 30 minutes at 4 ºC. Supernatant was then passed through a 3 kDa cutoff centrifugal filter (cellulose Amicon® , Merck) and dried under vacuum. Dry residues from shoots were dissolved in 20 µl of miliQ water, whereas root-and-nodule dry residues were dissolved in 12 µl. Then 5 µl aliquots were mixed with EDTA (final concentration 8.33 mM) to dissociate potential nicotianamine-metal complexes, and 50% (v/v) mobile phase A (see below) to favor chromatographic separation. The mixture was filtered through 0.45 polyvinylidene fluoride (PVDF) ultrafree-MC centrifugal filter devices (Merck) before analysis. Nicotianamine (Toronto Research Chemicals) calibration curve were prepared with nycotyl-lysine as internal standard.

Nicotianamine levels were determined by high-performance liquid chromatography electrospray ionization time-of-flight mass spectrometry (HPLC-ESI-TOF-MS) as described by Banakar et al. (2017). The samples were fractionated using an Alliance 2795 HPLC system (Waters) and µLC column (SeQuant ZIC®-HILIC, 15 cm x 1 mm internal diameter, 5 µm, 200 Å, Merck), with a mobile phase consisting of solvent A (9:1 10 mM ammonium acetate:acetonitrile, pH 7.3) and solvent B (8:2 30 mM ammonium acetate:acetonitrile, pH 7.3) at a flow rate of 0.15 mL min^{-1}. The gradient program started at 100% (v/v) solvent A for 3 min, and then decreased linearly to 30% (v/v) solvent A over the next 7 min, then remained for 7 min at 30% (v/v) solvent A, and then returned to the initial conditions over the next 8 min. The column was then allowed to stabilize for 10 min at the initial conditions before proceeding to the next injection. The total HPLC run time was 35 min, the injection volume was 10 µl and the auto sampler and column temperatures were 6 ºC and 30 ºC, respectively. The HPLC was coupled to the MicrOTOF mass spectrometer (Bruker Daltonics) equipped with an ESI source. The operating conditions were optimized by the direct injection of 100 µM solutions of nicotianamine standard at a flow rate of 180 µl h^{-1}. Mass spectra were acquired in negative ion mode over the 150–

700 mass-to-charge (*m/z*) ratio range. The mass axis was calibrated externally using Li–formate adducts (10 mM LiOH, 0.2% (v/v) formic acid and 50% (v/v) 2-propanol). Bruker Daltonik software packages micrOTOF Control v2.2, HyStar v3.2 and Data Analysis v4.0 were used to control the MS, HPLC interface and for data processing, respectively. Nicotianamine concentrations were quantified by external calibration, using nicotyl-lysine as internal standard.

1.3 References

Banakar R, Álvarez-Fernández A, Abadía J, Capell T, Christou P. (2017) The expression of heterologous Fe (III) phytosiderophore transporter *HvYS1* in rice increases Fe uptake, translocation and seed loading and excludes heavy metals by selective Fe transport. *Plant Biotechnol. J.* **15**, 423-432.

Supplementary Table 1. *Tnt1* gene insertions in *M. truncatula* NF15101 mutant line. N.R stands for non-reported.

Insertion	Gene ID	Homology to
high 1	N.R.	
high 2	N.R.	
high 3	*Medtr2g079280*	ubiquitin-conjugating enzyme
high 4	N.R.	
high 5	N.R.	
high 6	*Medtr5g023730*	fatty acyl-CoA synthetase family protein
high 7	*Medtr0400s0040*	LRR receptor-like kinase family protein
high 8	NOL1/NOP2/sun family protein	Coexpressed with genes in leaf specific coexpression subnetwork
high 9	N.R.	
high 10	N.R.	
high 11	*Medtr5g064610*	Thylakoid lumenal 29 kDa protein
high 12	N.R.	
high 13	*Medtr8g038400*	ADP-ribosylation factor GTPase-activating protein AGD10
high 14	*Medtr5g016060*	phototropic-responsive NPH3 family protein
high 15	*Medtr5g021860*	F-box protein interaction domain protein
high 16	N.R.	
high 17	N.R.	
high 18	N.R.	
high 19	*Medtr2g095800*	xyloglucan endotransglucosylase/hydrolase family protein
high 20	*Medtr2g070310*	MtNAS2
high 21	N.R.	
high 22	N.R.	

Supplementary Table 2. Primers used in this study

Name	Sequence	Use
Fw MtNAS2P- GW	GGGGACAAGTTTGTACAAAAAAGCAGGCTCTAA AATTGACAATTGTTATCA	Cloning of *MtNAS2* promoter in Gateway vector
Rv MtNAS2P-GW	GGGGACCACTTTGTACAAGAAAGCTGGGTATGG ATTGAATTGAAGTAATTT	Cloning of *MtNAS2* promoter in Gateway vector
Rv MtNAS2 GW	GGGGACCACTTTGTACAAGAAAGCTGGGTAAGCT TTAGGTTTGCGAAGAAG	Cloning of *MtNAS2* gene in Gateway vector
Fw MtNAS2	TGAAATTCCACAAGAGCTTCTCA	qRT-PCR of *MtNAS2*
Rv MtNAS2	TGGATGAAGGGAGGATGCAAA	qRT-PCR of *MtNAs2*
Fw MtUBQ9	GGTTGATTGCTCTTCTCTCCCC	qRT-PCR of gene encoding for ubiquitin conjugating enzyme E2
Rv MtUBQ9	AAGTGATTGCTCGTCCAACCC	qRT-PCR of gene encoding for ubiquitin conjugating enzyme E2

SUPPLEMENTARY FIGURE 1

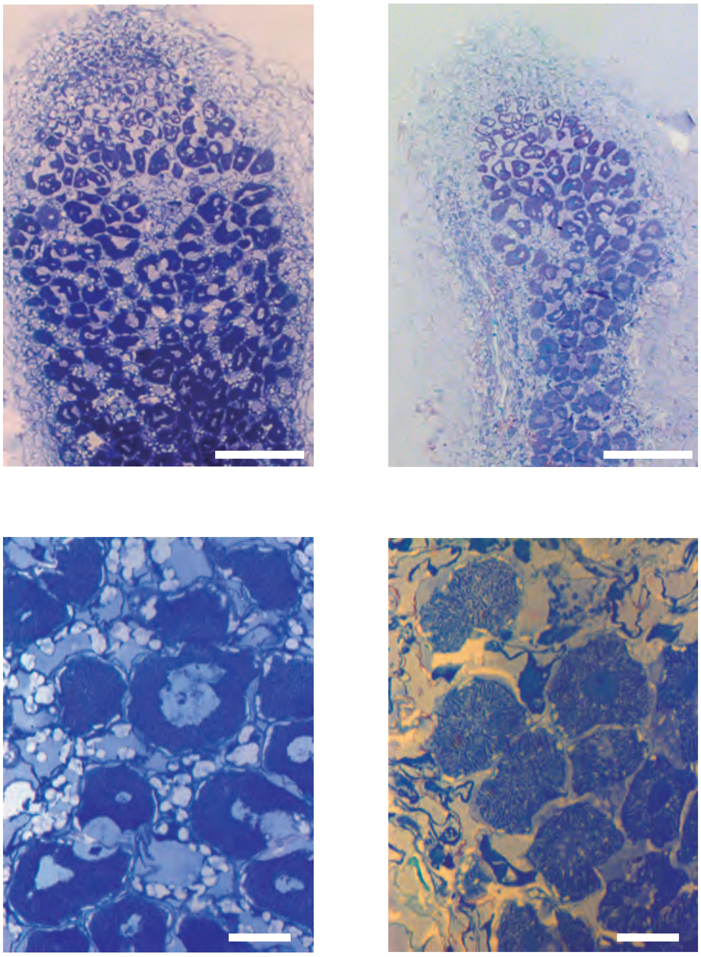

Supplementary Figure 1. Anatomy of 28 days-post-inoculation (dpi) wild type (left panels) and *nas2-1* (right panels) nodules. Toluidine blue stain of sections of 28 WT and *nas2-1* nodules. Bars = 50 µm (top panels) or 20 µm (lower panels).

SUPPLEMENTARY FIGURE 2

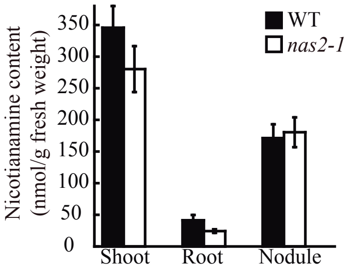

Supplementary Figure 2. Nicotianamine concentration in wild type (WT) and *nas2-1* 28 dpi plants. Data are the mean ± SE of n = 8-11 samples from three independent experiments.

SUPPLEMENTARY FIGURE 3

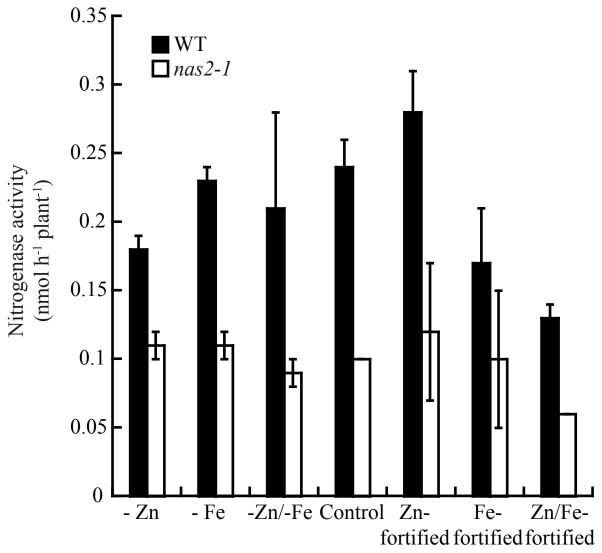

Supplementary Figure 3. Nitrogenase activity of wild type (WT) and *nas2-1* 28 dpi plants grown under symbiotic conditions when watered with the standard Jenner's solution (control), when no zinc and/or iron were added to the nutrient solution (-Zn, -Fe), or when the solution was fortified with 100x zinc (Zn-fortified), 0.5 g/l iron chelator sequestrene (Fe-fortified) or a combination of both. Acetylene reduction was measured in duplicate from three sets of three-four pooled plants. Data are the mean ± SE.

SUPPLEMENTARY FIGURE 4

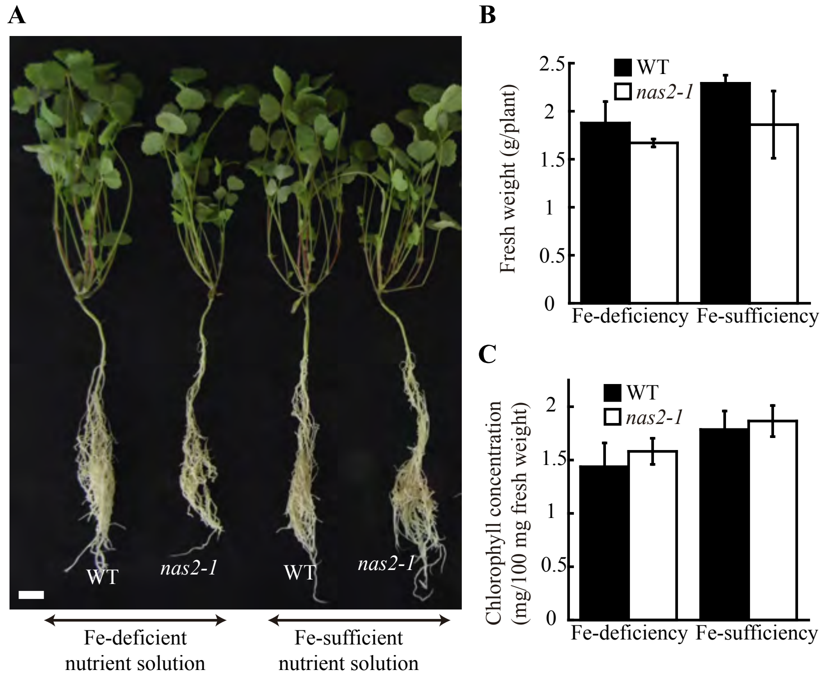

Supplementary Figure 4. Phenotype of *nas2-1* plants under low iron conditions. (A) Growth of representative wild type (WT) and *nas2-1* plants when watered with a nutrient solution supplemented with ammonium nitrate and not inoculated with S. meliloti. Bar = 1.5 cm. (B) Fresh weight of WT and *nas2-1* plants. Data are the mean ± SE of at least 5 plants. (C) Chlorophyll concentration of wild type and *nas2-1* plants. Data are the mean ± SE of two sets of 5 pooled plants.

SUPPLEMENTARY FIGURE 5

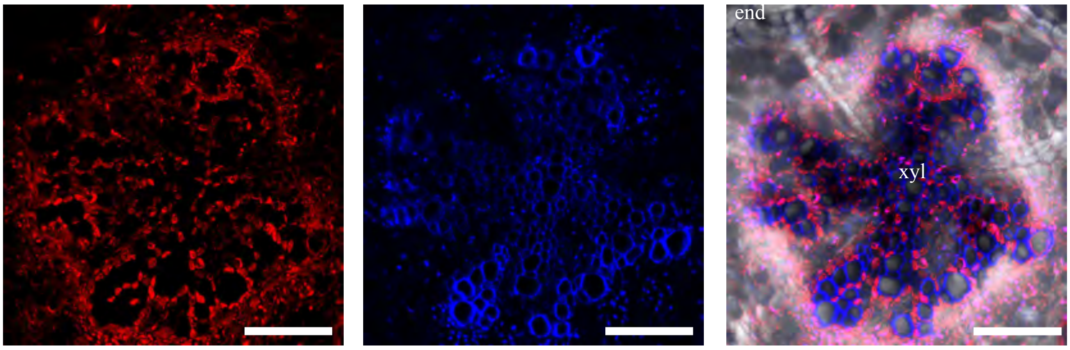

Supplementary Figure 5. Cross section of a a 28 dpi *M. truncatula* root expressing *MtNAS2-HA* under its own promoter. Left panel corresponds to the Alexa594 signal used to detect the HA-tag, middle panel corresponds to autofluorescence signal of xylem, and the two were overlaid with the bright field channel in the right panel. end indicates endodermis, and xyl, xylem. Bars = 100 µm.

SUPPLEMENTARY FIGURE 6

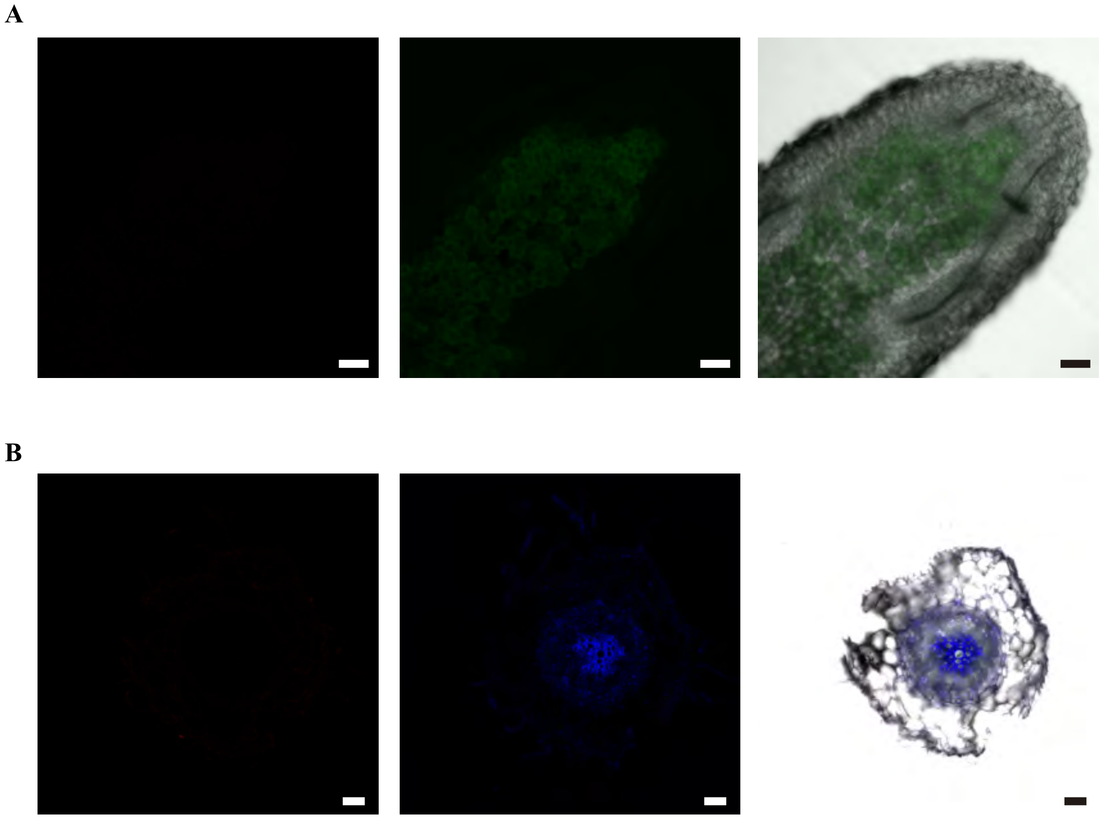

Supplementary Figure 6. Control for immunolocalization assays. (A) Longitudinal section of a 28 days-post-inoculation (dpi) *M. truncatula* nodule expressing *MtNAS2-HA* under its own promoter. Sections were treated as indicated for confocal microscopy, but without adding the Alexa594 conjugated antibody. The left panel corresponds to the Alexa594 emission signal. Transformed plants were inoculated with a GFP-expressing *S. meliloti* (green, middle panel). Both channels were overlaid with the transillumination image (right panel). Bars = 100 µm. (B). Cross section from a 28 dpi *M. truncatula* root expressing *MtNAS2-HA* under its own promoter. Left panel corresponds to the Alexa594 signal used to detect the HA-tag, middle panel corresponds to autofluorescence signal of xylem, and the two were overlaid with the bright field channel in the right panel. Bars = 50 µm.
